# Truncated PPM1D Prevents Apoptosis in the Murine Thymus and Promotes Ionizing Radiation-Induced Lymphoma

**DOI:** 10.3390/cells9092068

**Published:** 2020-09-10

**Authors:** Andra S. Martinikova, Monika Burocziova, Miroslav Stoyanov, Libor Macurek

**Affiliations:** 1 Laboratory of Cancer Cell Biology, Institute of Molecular Genetics of the Czech Academy of Sciences, Videnska 1083, CZ14220 Prague, Czech Republic; andra.vieru@img.cas.cz (A.S.M.); monika.burocziova@img.cas.cz (M.B.); miroslav.stoyanov@img.cas.cz (M.S.); 2 Department of Developmental and Cell Biology, Faculty of Science, Charles University, Albertov 6, CZ12800 Prague, Czech Republic

**Keywords:** cell-cycle checkpoint, tumor suppressor p53, protein phosphatase, cancer

## Abstract

Genome integrity is protected by the cell-cycle checkpoints that prevent cell proliferation in the presence of DNA damage and allow time for DNA repair. The transient checkpoint arrest together with cellular senescence represent an intrinsic barrier to tumorigenesis. Tumor suppressor p53 is an integral part of the checkpoints and its inactivating mutations promote cancer growth. Protein phosphatase magnesium-dependent 1 (PPM1D) is a negative regulator of p53. Although its loss impairs recovery from the G2 checkpoint and promotes induction of senescence, amplification of the *PPM1D* locus or gain-of-function truncating mutations of *PPM1D* occur in various cancers. Here we used a transgenic mouse model carrying a truncating mutation in exon 6 of *PPM1D* (*Ppm1d*^T^). As with human cell lines, we found that the truncated PPM1D was present at high levels in the mouse thymus. Truncated PPM1D did not affect differentiation of T-cells in the thymus but it impaired their response to ionizing radiation (IR). Thymocytes in *Ppm1d^T/+^* mice did not arrest in the checkpoint and continued to proliferate despite the presence of DNA damage. In addition, we observed a decreased level of apoptosis in the thymi of *Ppm1d^T/+^* mice. Moreover, the frequency of the IR-induced T-cell lymphomas increased in *Ppm1d^T/+^Trp53^+/−^* mice resulting in decreased survival. We conclude that truncated PPM1D partially suppresses the p53 pathway in the mouse thymus and potentiates tumor formation under the condition of a partial loss of p53 function.

## 1. Introduction

In healthy cells, genome stability is protected by cell-cycle checkpoints that prevent cell proliferation in the presence of DNA damage and allow time for DNA repair [1,2,3]. Induction of DNA double-strand breaks (DSBs, such as the ones generated by ionizing radiation (IR)) or the presence of structural chromosomal aberrancies emanating from mitotic defects activate ATM kinase that phosphorylates the tumor suppressor protein p53, which leads to its stabilization and transcriptional activation [4,5,6]. By triggering the expression of CDKN1A^p21^, p53 induces cell-cycle arrest in the G1 checkpoint [7]. Replication stress (caused by various factors including oncogene activation) leads to activation of the Ataxia telangiectasia and Rad3-related (ATR) kinase that triggers phosphorylation of the checkpoint kinase CHK1 that induces a cell-cycle arrest in the S/G2 phase by targeting CDC25 phosphatases [8]. Maintenance of the G2 checkpoint is then promoted by activation of the p53 pathway [9]. Recovery from the G2 checkpoint depends on protein phosphatase magnesium-dependent 1 delta (PPM1D, also called WIP1) that counteracts the activity of ATM at chromatin and prevents full activation of p53 [10,11,12,13]. Sustained activation of p53 during G2 can lead to premature activation of Anaphase-promoting complex/cyclosome and its coactivator Cdh1 followed by degradation of cyclin B1 and induction of senescence [14,15]. The depletion of PPM1D leads to abnormally high activation of p53 during G2 and to defects in homologous recombination, both contributing to the loss of recovery competence and induction of senescence [10,16,17]. In addition, senescence can result from sustained activation of p53 during G1 through the expression of downstream genes including CDKN1A^p21^ [18,19]. PPM1D is not required for terminating the G1 checkpoint, as other phosphatases (including PP4) can suppress the p53 response after completion of DNA repair [20]. Importantly, the checkpoint pathway and oncogene-induced senescence represent an intrinsic barrier preventing genome instability and tumor development [21,22,23].

Amplification of the chromosomal locus 17q23.2 carrying the *PPM1D* is common in various cancer types and results in overexpression of enzymatically active PPM1D [24]. Amplification of *PPM1D* has been reported in about 10% of breast cancers, mainly those that retain a wild-type p53 status [24,25,26]. Data from *PPM1D* knock-out mice demonstrate that PPM1D promotes tumor growth by inhibiting p53 and p38/MAPK pathways [24,27,28]. In addition, high expression of PPM1D can also affect response to therapy as it reduces the sensitivity of cancer cells to doxorubicin and other chemotherapeutics [29,30]. Recently, we and others described new pathogenic mutations in exon 6 of the *PPM1D* that result in production of the C-terminally truncated PPM1D protein [31,32,33]. The deletion of the last 60 amino acids of PPM1D removes a degron regulating its rapid turnover and results in stabilization of the truncated PPM1D protein [31,34]. Importantly, deletion of the C-terminal tail leaves the catalytic domain of PPM1D intact and also preserves chromatin localization of the truncated protein [31]. Cancer cell lines (including U2OS and HCT116 cells) carrying heterozygous truncating mutations in *PPM1D* show G1 checkpoint override upon exposure to the mild level of IR [31]. Similarly, when we introduced truncating mutations in exon 6 of the *PPM1D* in human non-transformed retinal pigment epithelial (RPE1) cell lines using CRISPR/Cas9 technology, we observed decreased ability to induce the G1 checkpoint after exposure to IR [35]. However, whether the truncating PPM1D mutations contribute to tumorigenesis remains an open question. To experimentally address this, we have recently generated a mouse model in which we introduced a frame-shift mutation in the exon 6 of *PPM1D* using the Transcription activator-like effector nuclease (TALEN) technology [35]. We have found that the truncated *PPM1D^T^* allele protected intestinal stem cells from apoptosis by suppressing the p53 pathway [35]. In addition, *PPM1D^T/+^* mice showed a higher amount of intestinal polyps and increased frequency of colon adenocarcinoma induced by constitutively active Wnt signaling in *APC^min^* background [35]. Although the *PPM1D^T^* allele alone did not induce the formation of colon tumors, it significantly potentiated the phenotype and reduced the survival of *APC^min^* mice [35].

T-cells differentiate in the thymus cortex from early progenitors by sequentially progressing through CD4^−^CD8^−^ double-negative (DN) and CD4^+^CD8^+^ double-positive (DP) stages and leave the medulla as single positive CD4+ or CD8+ cells with a fully assembled T-cell receptor (TCR) [36,37]. Site-specific dsDNA breaks in *TCR* gene present in DN cells trigger the p53 response and are eventually repaired by V(D)J recombination allowing the transition to the DP stage [38,39,40]. Sustained activation of p53 in mice lacking PPM1D blocked the T-cell maturation at the DN stage [41]. In addition, PPM1D has recently been implicated in maturation of the medullary thymic epithelial cells as well as in the development of the B cells [42,43,44].

Here we used the knock-in *Ppm1d^T^* mouse model to study the impact of truncated PPM1D on cell survival and tumorigenesis in murine thymus. We find that thymocytes carrying truncated PPM1D escape apoptotic cell death and continue proliferation despite the presence of DNA damage. Although the truncated PPM1D did not significantly drive tumorigenesis upon exposure of mice to IR, it promoted the formation of thymic lymphoma in *Trp53^+/−^* heterozygotes. We propose that truncation of PPM1D prevents full activation of p53 upon genotoxic stress and promotes tumor formation in cells exhibiting partial loss of p53.

## 2. Materials and Methods

### 2.1. Animals

All animal experiments were approved by the ethical committee of the Institute of Molecular Genetics (c.j. 1/2016) and were performed in C57Bl/6 mice. The mouse strain carrying a *Ppm1d*^T^ allele with a premature stop codon in exon 6 of the *Ppm1d* was described previously [35]. *Trp53^tm1Tyj/J^* mouse strain was obtained from the Jackson Laboratory (stock #002101) and was described previously [45]. Where indicated, mice were irradiated at age of 8–10 weeks using an X-RAD 225XL instrument equipped with a copper filter. Animal tissues were collected into a reagent for RNA isolation - RNA Blue (Top-Bio), homogenized using TissueLyser LT (Qiagen) and mixed with chloroform. RNA was isolated from the aqueous phase by precipitation with isopropanol. Proteins were isolated from the phenol-chloroform phase by precipitation with isopropanol, and after washing with 0.3 M guanine hydrochloride in 95% ethanol, proteins were dissolved in 2X Sodium Dodecyl Sulfate (SDS)-PAGE buffer.

### 2.2. Western Blotting

Protein concentration was measured by the Bicinchoninic Acid (BCA) protein assay (Thermo Fisher Scientific, Rockford, IL, USA). Proteins (30 μg) were separated on 4–20% gradient gels using SDS-PAGE and blotted onto a nitrocellulose membrane. Membranes were blocked in 3% milk in phosphate-buffer saline (PBS) for 2 h and incubated overnight with primary antibodies at 4 °C. The following antibodies were used for immunoblotting: rabbit monoclonal to PPM1D/WIP1 (Cell Signaling, #11901, clone D4F7), p53-pS15 (Cell Signaling, #12571), p21 (Abcam, #109199), KAP1-pS824 (Novus Biologicals, #NB100-2350), γH2AX-pS319 (Cell Signaling, #9718), p53 (Leica-CM5P-L), H2AX (Millipore, 07-627), Cleaved Caspase-3-Asp175 (Cell Signaling, #9664, clone 5A1E), KAP1 (GeneTex, Irvine, CA, USA, #102227, clone N1N2) and Importin β (Santa Cruz, sc-137016). After the washing, membranes were incubated with HRP-conjugated secondary antibodies (BioRad) and signal was detected using Enhanced chemiluminiscence (ECL) Western Blotting Substrate (Thermo Fisher Scientific, Rockford, IL, USA).

### 2.3. Flow Cytometry

Analysis of the developing T-cells by flow cytometry was performed as described [37]. Briefly, thymi from three 8-week old mice per genotype were smashed, strained and washed with 3% FBS and 2 mM EDTA. The mixture was centrifuged (300 g, 10 min) and red blood cells were lysed with the Ammonium-Chloride-Potassium (ACK) buffer (containing 150 mM NH4Cl, 10 mM KHCO3, and 0.1 mM EDTA) for 2 min. The lysis was stopped, the cell mixture was spun down and the pellet was stained with the following antibody cocktail: CD25-PE/Cy7 (Biolegend, San Diego, CA, USA, PC61 #102016), CD4-FITC (eBioscience GK1.5 # 11-0041-82), CD3-PerCP-Cy5.5 (Biolegend, San Diego, CA, USA, 17A2), CD8a-PE (Biolegend, San Diego, CA, USA, 53-6.7) and CD44-APC (Biolegend, San Diego, CA, USA, IM7) and Hoechst 33,258 (Sigma). Cells were gated using FSC/SSC, singlets were discriminated using SSC-W parameter following exclusion of dead cells positive for Hoechst staining. Developing T-cells were gated according to CD8 and CD4 markers, and the DN population was further separated based on the CD44 and CD25 positivity.

### 2.4. Immunohistochemistry

Tissues embedded in paraffin were sectioned on a Leica RM2255 microtome at a thickness of 3 μm. To examine the morphological changes in the tissues, the standard hematoxylin and eosin (H&E) staining protocol was followed. For staining with the Ki-67 proliferation marker (GeneTex, Irvine, CA, USA, #16667) and the Cleaved Caspase-3-Asp175 apoptotic marker (Cell Signaling, #9664, clone 5A1E), tissues were deparaffinized with xylene, isopropanol, and decreasing concentrations of ethanol. Heat-induced epitope retrieval was performed in 10 mM citrate buffer (pH 6.0). Samples were blocked with 0.3% hydrogen peroxide in methanol and in bovine-serum albumin (1%) with goat serum (5%) for 2h for Ki-67 staining or 5% BSA and 0.1% TX-100 in PBS for cleaved caspase staining. Incubation with the primary antibody was done overnight at 4 °C and with the biotin-conjugated secondary antibody for 1 h at room temperature. Signal was developed using VECTASTAIN ABC HRP kit (Vector Laboratories) and 3,3′-Diaminobenzidine (DAB). Apoptosis was detected using Click-iT™ Plus terminal deoxynucleotidyl transferase (TUNEL) Assay (Thermo Fisher Scientific, Rockford, IL, USA). Briefly, tissues were deparaffinized, fixed with 4% formaldehyde, and permeabilized with Proteinase K. The TdT reaction with Ethynyl-2′-Deoxyuridine, 5′-Triphosphate (EdUTP) incorporation into the dsDNA breaks was performed for 60 min at 37 °C. Click-iT™ Plus reaction for detecting EdUTP with Alexa Fluor 594 picolyl azide was performed for 30 min and nuclei were stained using 4′,6-Diamidino-2-Phenylindole, Dihydrochloride (DAPI). Slides were mounted using Q Path Coverquick mounting medium and visualized on a Leica DM6000 microscope (Leica) equipped with HC PL FLUOTAR 5X, and HC PLAN APO 20X and 40X objectives. Images were analyzed using ImageJ software [46]. After thresholding, total areas of positive and negative signal were measured in each image.

### 2.5. Real-Time Quantitative Reverse Transcriptase PCR (RT-qPCR)

cDNA was generated from 0.5 μg RNA using random hexamers and RevertAid Reverse Transcriptase (Thermo Fisher Scientific, Rockford, IL, USA). RT-qPCR was performed using LightCycler 480 SYBR Green I Master mix (Roche) as described [35]. The following primers were used for RT-qPCR: *CDKN1A/p21* TGAGGAGGAGCATGAATGGAGACA and AACAGGTCGGACATCACCAGGATT; *PUMA* CCT-GGAGGGTCATGTACAATCT and TGCTACATGGT-GCAGAAAAAGT; *PPM1D* AGCCAGGAG-ACCTGTGTGAT and GGCATTACTGCGAACAAGGG; *GAPDH* ACAGCCGCATCTTCTTGTGCA-GTG and GGCCTTGACTGTGCCGTTGAATTT. The LightCycler480 software was used to determine Ct values. The data is shown as a ratio between the tested mRNA and GAPDH mRNA.

### 2.6. Statistical Analysis

Several animals used in each experimental group is indicated. Immunoblots were performed using tissues from at least 3 animals and representative images are shown. Statistical significance was evaluated using Graph Pad Prism 5.04 software. Unless stated otherwise, two-tailed unpaired *t*-test was used and *p* values < 0.05 are considered statistically significant. Kaplan-Meier survival plot was evaluated using a log-rank test. Error bars indicate standard deviation.

## 3. Results

### 3.1. Mice with Truncated PPM1D Show Impaired Acute *DNA-Damage* Response (DDR) in the Thymus upon γ-Irradiation

**Figure 1:**
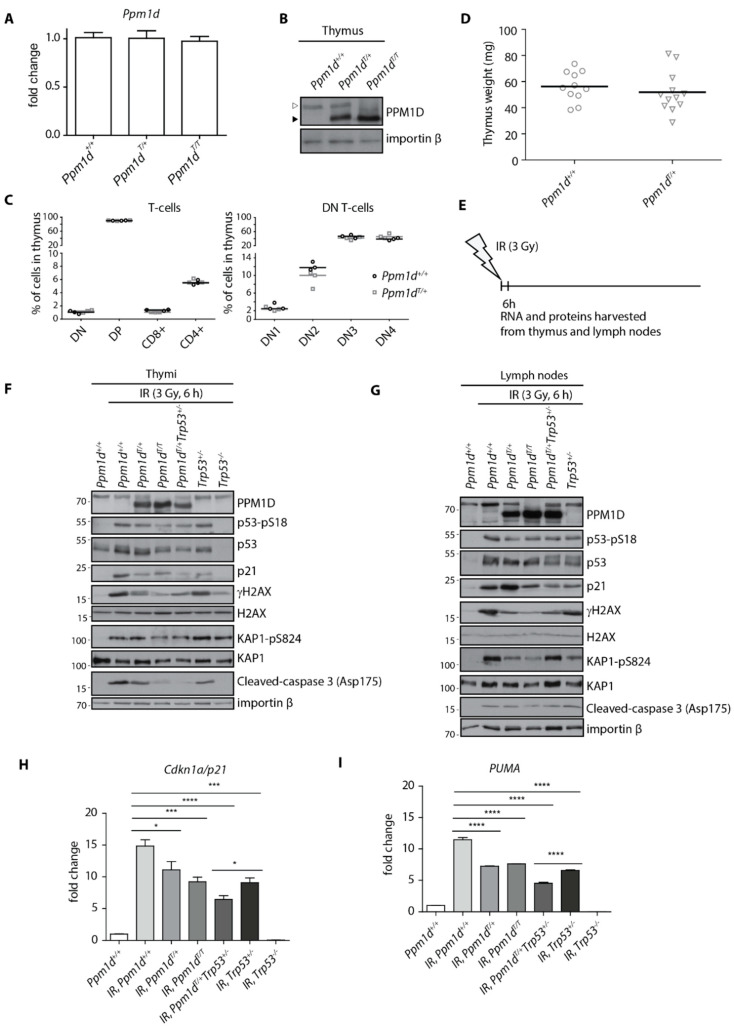
Truncated PPM1D impairs DNA damage response in mouse thymus. Expression of PPM1D mRNA was analyzed by RT-qPCR in the thymi of Ppm1d^+/+^, Ppm1d^T/+^ and Ppm1d^T/T^ mice and was normalized to GAPDH (*n* = 3) (**A**). Thymi from mice of indicated genotypes were lysed and proteins were separated by SDS-PAGE. Samples were probed with antibody against PPM1D and importin-β as a loading control. The empty and full arrowheads indicate the position of the full-length and the C-terminally truncated PPM1D, respectively. (**B**). Cells from thymi from *Ppm1d*^+/+^ and *Ppm1d*^T/+^ mice were analyzed by flow cytometry. Plotted are the counts of the indicated populations as follows: double-negative T-cells (DN and DN1, DN2, DN3, DN4), double-positive T-cells (DP), CD8-positive T-cells (CD8+) and CD4-positive T-cells (CD4+) (*n* = 3) (**C**). The median size of the thymus was determined in *Ppm1d ^+/+^* (*n* = 11) and *Ppm1d^T/+^* (*n* = 12) mice (**D**). A scheme of the experimental setup in F-I. Mice were exposed or not to a low dose of IR (3 Gy), sacrificed after 6 h and thymi and lymph nodes were collected (**E**). Proteins isolated from thymi from mice of indicated genotypes exposed to mock or to IR were probed with the indicated antibodies by immunoblotting (**F**). Proteins isolated from inguinal lymph nodes from mice of indicated genotypes exposed to mock or to IR were probed with the indicated antibodies by immunoblotting (**G**). RNA isolated from thymi from mice in E was analyzed by RT-qPCR. The expression of *CDKN1A^p21^* mRNA was normalized to *GAPDH*. Statistical significance was evaluated by two-tailed *t*-test, error bars indicate SD, *n* = 5 (**H**). RNA isolated from thymi from mice in D was analyzed by RT-qPCR. The expression of *PUMA* mRNA was normalized to *GAPDH*. Statistical significance was evaluated by two-tailed *t*-test, error bars indicate SD, *n* = 5. * *p* <0.05; *** *p* < 0.0005; **** *p* < 0.0001 (**I**).

Previous reports described PPM1D expression in the developing thymocytes, fully differentiated T-cells and medullar thymic epithelial cells [41,42]. The loss of *Ppm1d* blocked progression of thymocytes from a DN to DP stage resulting in a decreased population of differentiated T-cells and in reduction of the thymus size in *Ppm1d^−/−^* mice [41]. As the block in T-cell development was associated with an increased activation of the tumor suppressor p53, we aimed here to evaluate the impact of PPM1D truncation on T-cell maturation using the *PPM1D^T^* knock-in mouse model [41]. First, we measured the expression of Ppm1d in mouse thymi using RT-qPCR and found that the wild-type *Ppm1d* and truncated *Ppm1d^T^* were expressed at comparable levels suggesting that introducing a stop codon in the last exon does not affect transcriptional control of Ppm1d mRNA (Figure 1A). As expected, the truncated Ppm1d^T^ protein migrated faster on SDS-PAGE and was present at high levels due to stabilization of the truncated protein (Figure 1B) [31,35]. Using flow cytometry, we quantified individual populations of the developing T-cells in the thymi, but we did not find any significant differences between the wild-type and *Ppm1d*^T/+^ animals (Figure 1C). Consistent with this finding, we also did not observe any difference in the thymus size in the wild-type and *Ppm1d*^T/+^ animals (Figure 1D). These findings suggest that the truncated PPM1D does not affect the differentiation of the T-cells.

Next, we aimed to compare the response to genotoxic stress in lymphocytes carrying the wild-type PPM1D and the truncated PPM1D^T^. To this end, we exposed or not *Ppm1d*^+/+^, *Ppm1d*^T/+^ and *Ppm1d*^T/T^ animals to a low level of IR (3 Gy) and collected the thymi and lymph nodes after 6 h (Figure 1E). *Trp53^−/−^* animals lacking the p53 and *Trp53^+/−^* heterozygotes were analyzed in parallel as controls. As expected, wild-type animals showed increased phosphorylation of p53 at S18 (corresponding to the human S15) and induction of the p53 transcriptional target CDKN1A^p21^ after exposure to IR (Figure 1F). In contrast, PPM1D^T/+^ heterozygotes showed decreased level of p53.

Phosphorylation and a lower level of CDKN1A^p21^ induction (Figure 1F). Levels of CDKN1A^p21^ were comparable in *Ppm1d*^T/+^ and *Trp53^+/−^* whereas further reduction of CDKN1A^p21^ was observed in the thymi of *Ppm1d*^T/+^*Trp53^+/−^* double heterozygotes suggesting that truncated PPM1D can promote the phenotype of a partial loss of the p53. Similarly, truncation of both alleles in *Ppm1d*^T/T^ animals slightly enhanced dephosphorylation of p53-S18, *γ*-H2AX and KAP1-S824, all the established substrates of PPM1D, compared to *Ppm1d*^T/+^ but surprisingly we did not observe further reduction of p21 levels (Figure 1F, Supplementary Figure S1A,B). Probing for a cleaved caspase 3 suggested that even the mild dose of IR can lead to activation of the apoptotic pathway in wild-type thymocytes (Figure 1F). Importantly, activation of the apoptotic pathway was p53-dependent as cleavage of the caspase 3 did not occur in *Trp53^−/−^* animals. Notably, we observed a lower level of cleaved caspase 3 in the thymi from *Ppm1d*^T/+^ and *Trp53^+/−^* animals and further reduction in *Ppm1d*^T/+^*Trp53^+/−^* double heterozygotes (Figure 1F). The impact of the truncated PPM1D on the expression of *CDKN1A^p21^* and a pro-apoptotic gene *PUMA* was further confirmed by a RT-qPCR (Figure 1H,I). In good agreement with the results above, we observed a reduced expression of *CDKN1A ^p21^* and *PUMA* in the thymi from *Ppm1d*^T/+^*Trp53^+/−^* double heterozygotes compared to *Ppm1d*^T/+^ and *Trp53^+/−^* animals.

In parallel with the thymi, we analyzed the response to genotoxic stress in the lymph nodes (Figure 1G, Supplementary Figure S1C,D). As expected, we observed decreased level of *γ*-H2AX in mice carrying truncated PPM1D. Reduction of KAP1 phosphorylation was even more prominent in the lymph nodes than in the thymi confirming that a high activity of PPM1D was present in cells. Surprisingly, we did not observe any reduction of p21 and cleaved caspase 3 levels in mice carrying *PPM1D^T^* allele suggesting that its impact on the cell-cycle checkpoint and apoptosis may differ in various cell types and tissues.

We conclude that the acute response to genotoxic stress is partially suppressed by truncated PPM1D in the murine thymi, which is in good agreement with our recent observation of the impaired signaling in the intestinal stem cells in mice carrying *Ppm1d*^T^ allele [35].

### 3.2. Truncated PPM1D Prevents Apoptosis and Provides a Proliferation Advantage after Genotoxic Stress

**Figure 2:**
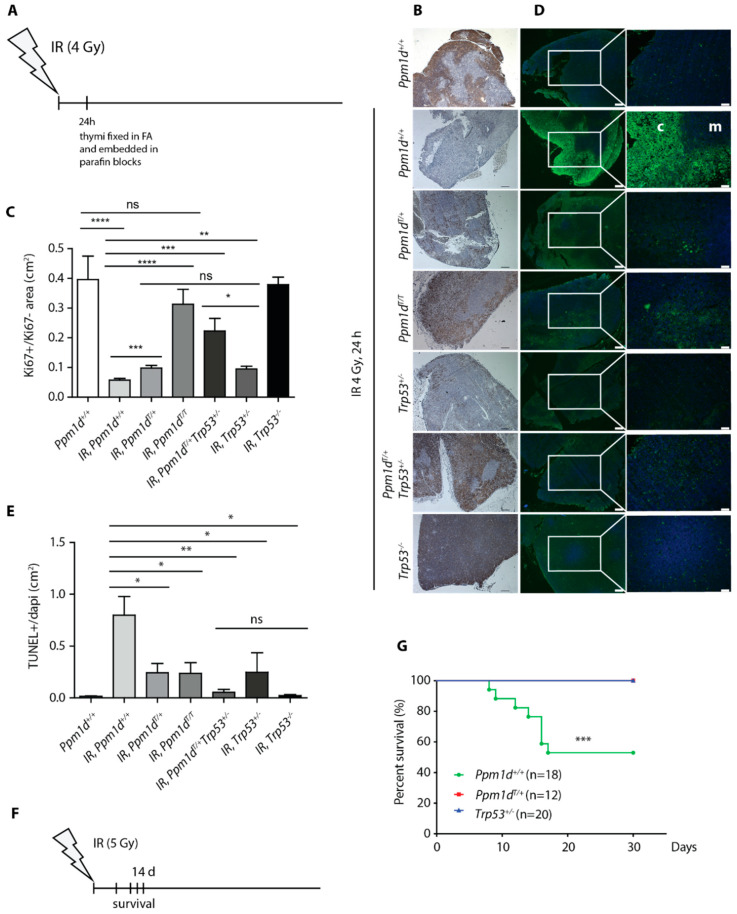
Truncated PPM1D prevents apoptosis and promotes proliferation after genotoxic stress. A scheme of the experimental setup of B–E. Thymi were collected from mice sacrificed 24 h after exposure to mock or to IR (4 Gy) and analyzed by immunohistochemistry (**A**). Histology sections of thymi from mice of indicated genotypes exposed to mock or to IR (4 Gy) were probed with antibody against Ki-67 (proliferation marker). Representative images are shown. Magnification 5×, bars indicate 200 μm (**B**). Quantification of Ki-67 signal from B. At least 3 sections from 3 mice per genotype were quantified. Error bars indicate SD. Statistical significance was evaluated by two-tailed *t*-test, * *p* < 0.05; ** *p* < 0.01; *** *p* < 0.0005; **** *p* < 0.0001. (**C**). Histology sections of thymi from mice of indicated genotypes exposed to mock or to IR were subjected to TUNEL assay (apoptosis marker). Note high TUNEL+ signal in cortex (c) but not in medulla (m) of the *Ppm1d*^+/+^*Trp53^+/+^* mice. Representative images are shown. Magnification 5× and 20×, bars indicate 200 μm and 50 μm, respectively (**D**). Quantification of TUNEL+ signal from D. At least 3 sections from 3 mice per genotype were quantified. Error bars indicate SD. Statistical significance was evaluated by two-tailed *t*-test, * *p* < 0.05; ** *p* < 0.01 (**E**). A scheme of the experimental setup in G (**F**). Wild-type *Ppm1d*^+/+^*Trp53^+/+^*, *Ppm1d*^T/+^*Trp53^+/+^*, and *Ppm1d*^+/+^*Trp53^+/^^−^* mice were exposed to 5 Gy of IR and their survival was monitored for subsequent 30 days. *n* values indicate the numbers of animals of each genotype. Statistical significance of the Kaplan-Meier survival plot was determined by a log-rank test, **** *p* < 0.005 (**G**).

Decreased levels of CDKN1A^p21^ and of cleaved caspase-3 in the thymi of the *Ppm1d*^T/+^ mice compared to the wild-type animals suggested that truncated PPM1D may impair activation of the cell-cycle checkpoint and induction of apoptosis after exposure to IR. Consistent with the cell-cycle arrest, we observed dramatically reduced signal of the proliferation marker Ki-67 in the thymi from the irradiated (4 Gy) wild-type mice (Figure 2A–C). Under the same conditions, *Ppm1d*^T/+^ mice showed significantly higher staining of Ki-67 marker upon irradiation to a level comparable with *Trp53^+/^^−^* heterozygotes (Figure 2B,C, Supplementary Figure S2A). Further increase in the proliferation was observed in *Ppm1d*^T/+^*Trp53^+/^^−^* double heterozygotes and in *Ppm1d^T/T^* animals exposed to IR and the Ki-67 signal in these mice was comparable to the control wild-type animals that were not exposed to genotoxic stress (Figure 2B,C). We conclude that the proliferation of thymocytes in *Ppm1d*^T/+^ animals may continue despite the presence of DNA damage. This defect in the checkpoint is further increased in *Ppm1d*^T/T^ homozygotes suggesting that higher level of PPM1D may be needed to fully suppress the p53 pathway. Moreover, the intermediate phenotype observed in p53 heterozygotes is enhanced by truncation of one allele of the *Ppm1d*.

Next, we analyzed the same set of samples using TUNEL assay for DNA fragmentation, which is a hallmark of apoptotic cells [47,48]. Opposite to the Ki-67 signal, we observed high induction of the TUNEL+ signal in the thymic cortex of the wild-type animals 24 h after exposure to IR (Figure 2D,E, Supplementary Figure S2B). Notably, TUNEL+ signal was lower in *Ppm1d*^T/+^ and *Ppm1d*^T/T^ mice and was further reduced in *Ppm1d*^T/+^*Trp53^+/−^* double heterozygotes to the level comparable to *Trp53^−/−^* knock-out animals (Figure 2D,E). To confirm that occurrence of the TUNEL signal corresponded to induction of apoptosis, we stained the sections from the thymi for the cleaved caspase 3 (Supplementary Figure S3A,B). Indeed, we observed a strong activation of the caspase 3 in the wild-type mice at 6 h after irradiation, whereas the staining was reduced in *Ppm1d*^T/+^ animals suggesting that the truncated PPM1D impaired induction of the apoptosis (Supplementary Figure S3A,B). Finally, we analyzed the thymi from the control and irradiated mice by flow cytometry (Supplementary Figure S3C). Although the DP CD4+/CD8+ cells represented a major population in the thymi of the control mice, we observed a strong reduction of the CD4+/CD8+ cells upon irradiation. On the other hand, reduction of the CD4+/CD8+ population was significantly lower in *Ppm1d*^T/+^ animals suggesting that truncated PPM1D protected these cells from programmed cell death. Overall, our data are consistent with impaired induction of apoptosis in the thymi of mice carrying truncated PPM1D although we have not formally excluded the possibility that the T-cells escape cell death by reverting the late stage of apoptosis through a process called anastasis [49,50].

Depending on the dose of IR, cell death occurs in various tissues and a loss of the stem cells in the bone marrow and the gastrointestinal tract can lead to a fatal failure of these organs. As we observed decreased level of apoptosis in the thymi of the *Ppm1d*^T/+^ animals we hypothesized that these animals could be more resistant to IR. Indeed, we observed that about half of the wild-type mice died within 2 weeks upon exposure to a single dose (5 Gy) of whole-body irradiation (Figure 2F,G). In striking contrast, none of the *Ppm1d*^T/+^ mice died under these conditions confirming that truncated PPM1D efficiently suppresses cell death in various tissues (Figure 2G). Interestingly, *Trp53^+/^^−^* mice were also resistant to IR suggesting that the effect of truncated Ppm1d could be mediated by partial suppression of p53 function.

### 3.3. Truncated PPM1D Promotes Formation of the Ionizing Radiation-Induced Lymphoma

**Figure 3:**
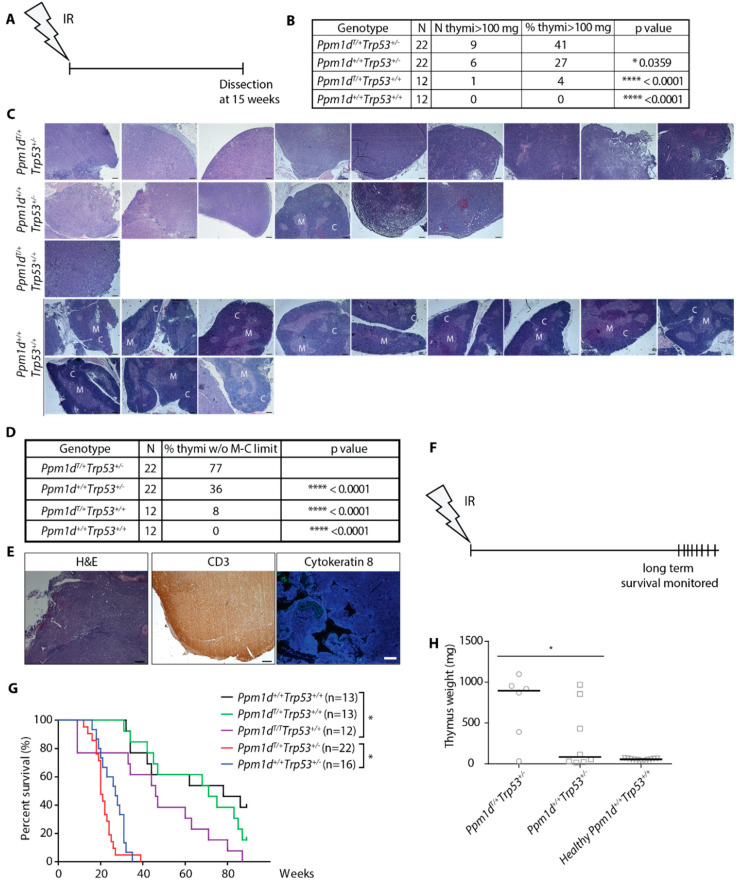
Truncated PPM1D promotes formation of IR-induced lymphoma in mice. Schematic of the experimental setup. At 8–10 weeks of age, mice were exposed to a single dose of ionizing radiation (4 Gy) and followed for another 15 weeks when they were sacrificed and subjected to autopsy. Thymi were evaluated by histology (**A**). Mice of indicated genotypes were treated as in A. Percentage of thymi of abnormal weight (>100 mg) was scored. *n* value indicates the number of animals in each experimental group. Statistical significance to the *Ppm1d*^T/+^*Trp53^+/^^−^* was determined by Fisher’s one-tailed exact test, * *p* < 0.05; **** *p* < 0.0001. (**B**). H&E staining of the enlarged thymi from mice in A and B. Representative images of thymi considered to be enlarged are shown for genotypes *Ppm1d*^+/*−*^, *Trp53^+/^^−^*, and *Ppm1d*^T/+^*Trp53^+/^^−^* mice and all thymi from the wild-type mice are shown. Regions of the cortex (C) and medulla (M) are indicated. Magnification 5×, scale bar indicates 200 µm (**C**). Quantification of delineation between cortex and medulla from the experiment in C. Multiple sections were evaluated to cover the whole thymus. Statistical significance to the *Ppm1d*^T/+^*Trp53^+/^^−^* was determined by Fisher’s one-tailed exact test, **** *p* < 0.0001. (**D**). Representative images of a thymic tumor from B are shown. Section of the thymus was stained with H&E, or an antibody against CD3 (T-cell marker) or cytokeratin 8 (stromal marker) (**E**). Schematic of the experimental setup in G-H (**F**). Mice of indicated genotypes were exposed to a single dose of IR (4 Gy) and their survival was followed in time. *n* values indicate the numbers of animals of each genotype. Statistical significance of the Kaplan-Meier survival plot was determined by a log-rank test, * *p* < 0.05. (**G**). Terminally moribund mice from G were sacrificed, dissected, and median size of thymi was determined. Statistical significance was determined by the one-tailed *t*-test (*p =* 0.045) (**H**).

Encouraged by a striking difference in the survival of *Ppm1d*^T/+^ and *Ppm1d*^+/+^ animals after exposure to a high dose of IR, we aimed to investigate the long-term consequences of the exposure to a sublethal dose. We hypothesized that continued proliferation in the presence of genotoxic stress may lead to genome instability and promote cancer development in animals carrying truncated Ppm1d. To test this possibility we employed a model of IR-induced thymic lymphoma that develops in mice with suppressed p53 pathway upon single exposure to a sublethal dose of ionizing radiation [51,52]. As expected, all wild-type animals (12 out of 12) showed normal thymus size (Figure 3A,B, Supplementary Figure S4A) and morphology defined by the medullo-cortical delimitation (Figure 3C,D) 15 weeks after exposure to 4 Gy. As expected, about one fourth (6/22) of the *Trp53^+/^^−^* mice showed increased thymus weight due to lymphoma development (Figure 3B, Supplementary Figure S4A).

Under the same conditions, one out of 12 *Ppm1d*^T/+^ animals had an enlarged thymus (Figure 3B, Supplementary Figure S4A) and showed impaired delineation between thymic medulla and cortex (Figure 3C,D) indicative of the tumor growth [53]. Notably, higher penetrance of the phenotype was observed in *Ppm1d*^T/+^*Trp53^+/^^−^* double heterozygotes out of which 41% (9/22) developed lymphoma (Figure 3B, Supplementary Figure S3A). Similarly, the loss of medulla/cortex delineation increased in the *Ppm1d*^T/+^*Trp53^+/^^−^* double heterozygotes compared to *Trp53^+/^^−^* mice (Figure 3C,D). Immunohistochemical staining for CD3 (a T-cell marker) and cytokeratin 8 (a marker of thymic epithelial cells, [54]) revealed that the thymic tumors were T-cell lymphomas (Figure 3E). In addition to the T-cell lymphoma, one of the *Ppm1d*^T/+^*Trp53^+/^^−^* mice with a normal thymus size developed a fibrosarcoma (Supplementary Figure S4B). Another *Ppm1d*^T/+^*Trp53^+/^^−^* mouse showed impaired medulla/cortex delineation in a thymus of a border size (80.2 mg vs. 62.8 mg median thymus weight in the wild-type animals) and enlarged kidney also referred to as hydronephros) that has previously been linked with T-cell lymphomas [55] (Supplementary Figure S4C). In summary, our data suggest that truncation of Ppm1d might slightly increase frequency of the thymic lymphoma development (1/12 compared to 0/12 tumors in the wild-type animals) although considerably larger animal groups would be required to evaluate the statistical significance. On the other hand, we observed a significantly increased number of the tumors in *Ppm1d*^T/+^*Trp53^+/−^* double heterozygotes compared to *Trp53^+/−^* indicating that truncated Ppm1d may promote tumorigenesis in cells with a partial loss of p53 function.

Finally, we compared survival of mice after single exposure to a sublethal dose of IR. We found that median survival of the wild-type mice was about 70 weeks and was reduced to 30 weeks in *Trp53^+/−^* heterozygotes (Figure 3F,G). Significant reduction of the survival to 20 weeks was observed in *Ppm1d*^T/+^*Trp53^+/−^* double heterozygotes which is consistent with the increased frequency of the tumors described above (Figure 3G). Under the same conditions, overall survival of *Ppm1d*^T/+^ was indistinguishable from survival of the wild-type animals. However, survival of *Ppm1d*^T/T^ homozygotes was reduced to 50 weeks which is consistent with a more pronounced defect in DNA damage response in *Ppm1d*^T/T^ homozygotes compared to *Ppm1d*^T/+^ heterozygotes (Figure 3G). Moribund mice were sacrificed, and subsequent autopsy confirmed the presence of a T-cell lymphoma in 83% of *Ppm1d*^T/+^*Trp53^+/−^* mice compared to 37% in *Trp53^+/−^* mice (Figure 3H). We conclude that truncated Ppm1d reduces the survival of mice with a partial loss of p53 due to the increased frequency of the T-cell lymphoma formation.

## 4. Discussion

**Figure 4:**
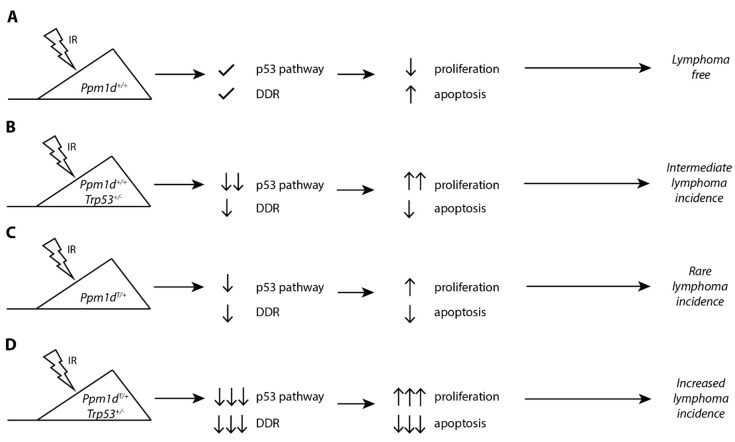
A proposed model for the role of truncated PPM1D in the ionizing radiation-induced lymphoma. Mice carrying wild-type *Ppm1d* and *Tpr53* activate the p53 pathway and DNA damage response upon exposure to ionizing radiation leading to block of the cell proliferation or to apoptosis in the thymus. This protects genome integrity and prevents tumor development (**A**). A partial loss of the p53 function in *Trp53^+/^^−^* mice suppresses activation of the checkpoint induced by ionizing radiation. This allows continuous cell proliferation in the presence of DNA damage leading to accumulation of the genetic changes and lymphoma development (**B**). Mice carrying C-terminally truncated *Ppm1d* fail to fully activate p53 and DNA damage response after exposure to ionizing radiation. In most cases, residual p53 function is sufficient to suppress proliferation and tumor formation. However, a fraction of cells proliferates despite the existing DNA damage which may occasionally result in tumor development (**C**). Combination of a partial loss of p53 and suppression of its function in *Ppm1d*^T/+^*Trp53^+/^^−^* mice allows continuous proliferation in the presence of DNA damage and prevents clearance of damaged cells by apoptosis. As a result, thymic lymphoma develops more frequently in *Ppm1d*^T/+^*Trp53^+/^^−^* mice compared to *Ppm1d*^T/+^ or *Trp53^+/^^−^* alone (**D**).

In this study, we exploited our recently generated transgenic model of the truncated Ppm1d to study its impact on DNA damage response in T lymphocytes and to evaluate its role in tumorigenesis (Figure 4). As with the mouse intestinal stem cells, we observe that the truncated Ppm1d accumulates in the T-cells at high quantities and impairs the cell response to genotoxic stress. Upon exposure to IR, cells carrying truncated Ppm1d express lower amount of *CDKN1A^p21^* and *PUMA*. As a result, the proliferation of the thymocytes in *Ppm1d*^T/+^ mice is not completely suppressed after exposure to IR which is consistent with a deficient checkpoint arrest that we previously reported in Human cells [31]. Moreover, clearance of damaged cells by a programmed cell death is also suppressed in the thymi of *Ppm1d*^T/+^ mice. Continuous cell proliferation and suppressed cell death under the condition of genotoxic stress are even more prominent in *Ppm1d*^T/+^*Trp53^+/^^−^* mice suggesting that truncation of Ppm1d suppresses p53 function only partially. In addition, we observed that the *Ppm1d*^T/T^ homozygote showed a stronger phenotype than *Ppm1d*^T/+^ heterozygote in terms of the suppression of p53 function. This finding is surprising considering that a homozygous *PPM1D* mutation has not been reported in human tumors so far. Cancer cell lines carrying truncated PPM1D (including U2OS and HCT116 cells) are heterozygotes, and also colon carcinoma samples do not contain both mutant alleles [31,35]. Multiple nonsense or frame-shift mutations in the *PPM1D* observed in Acute Myeloid Leukemia (AML) patients spread throughout the whole exon 6 which results in many variants of the PPM1D protein with differently a sized C-terminal tail [34,56]. When performing CRISPR/Cas9 mutagenesis at various positions in exon 6 of the PPM1D we observed that some of the truncated PPM1D variants exhibited higher protein stability than others (data not shown). It is plausible that a similar situation occurs in human tumors and that mutations of PPM1D resulting in higher protein stability may allow stronger suppression of p53 than mutations leading to lower PPM1D levels. Although the stability of Ppm1d in the mouse model is clearly increased and the truncated Ppm1d is present at a higher level than the full-length Ppm1d, we observe just a partial effect on suppression of p53 resulting in a relatively weak cancer phenotype. Slight increase of Ppm1d activity in the *Ppm1d*^T/T^ homozygote could suppress p53 function more efficiently leading to increased probability of tumor formation. We speculate that another Ppm1d truncation leading to higher protein stability could possibly reach the threshold required for efficient suppression of p53 already in a heterozygous state. It will be interesting to address this possibility in the future by generating other mouse models with nonsense mutations at various positions in exon 6 of the *Ppm1d*.

Amplification of the *PPM1D* locus leading to high expression levels of the full-length PPM1D protein is relatively common in human breast cancer and was observed mainly in tumors retaining the wild-type p53 [24,57]. Data from the transgenic mouse model used in this study suggest that the truncated Ppm1d has the potential to increase the pathogenicity of a partial loss of p53. Future research is needed to experimentally validate this possibility in clinically relevant human cancers.

## Supplementary Materials

The following are available online at https://www.mdpi.com/2073-4409/9/9/2068/s1, Figure S1: Truncated PPM1D impairs DNA damage response, Figure S2: Image thresholding for quantification of proliferation and apoptosis in thymus, Figure S3: Truncated PPM1D prevents activation of caspase 3 after genotoxic stress, Figure S4: Truncated PPM1D promotes tumorigenesis in p53^+/−^ background.
