# Supplementary material for: Truncated PPM1D Prevents Apoptosis in the Murine Thymus and Promotes Ionizing Radiation-Induced Lymphoma
Source: Cells. 2020 Sep 10;9(9):2068. doi: 10.3390/cells9092068 (PMC7565556; doi:10.3390/cells9092068)
Supplement: Supplementary file 1 [file cells-09-02068-s001.pdf]

cells

Truncated PPM1D prevents apoptosis in murine thymus and promotes ionizing radiation-induced lymphoma

Andra S. Martinikova[1,2,#], Monika Burocziova[1,#], Miroslav Stoyanov, Libor Macurek[1]

[1] Laboratory of Cancer Cell Biology, Institute of Molecular Genetics of the Czech Academy of Sciences, Prague, Czech Republic

[2] Department of Developmental and Cell Biology, Faculty of Science, Charles University, Prague, Czech Republic

Supplementary file containing Figures 1-4

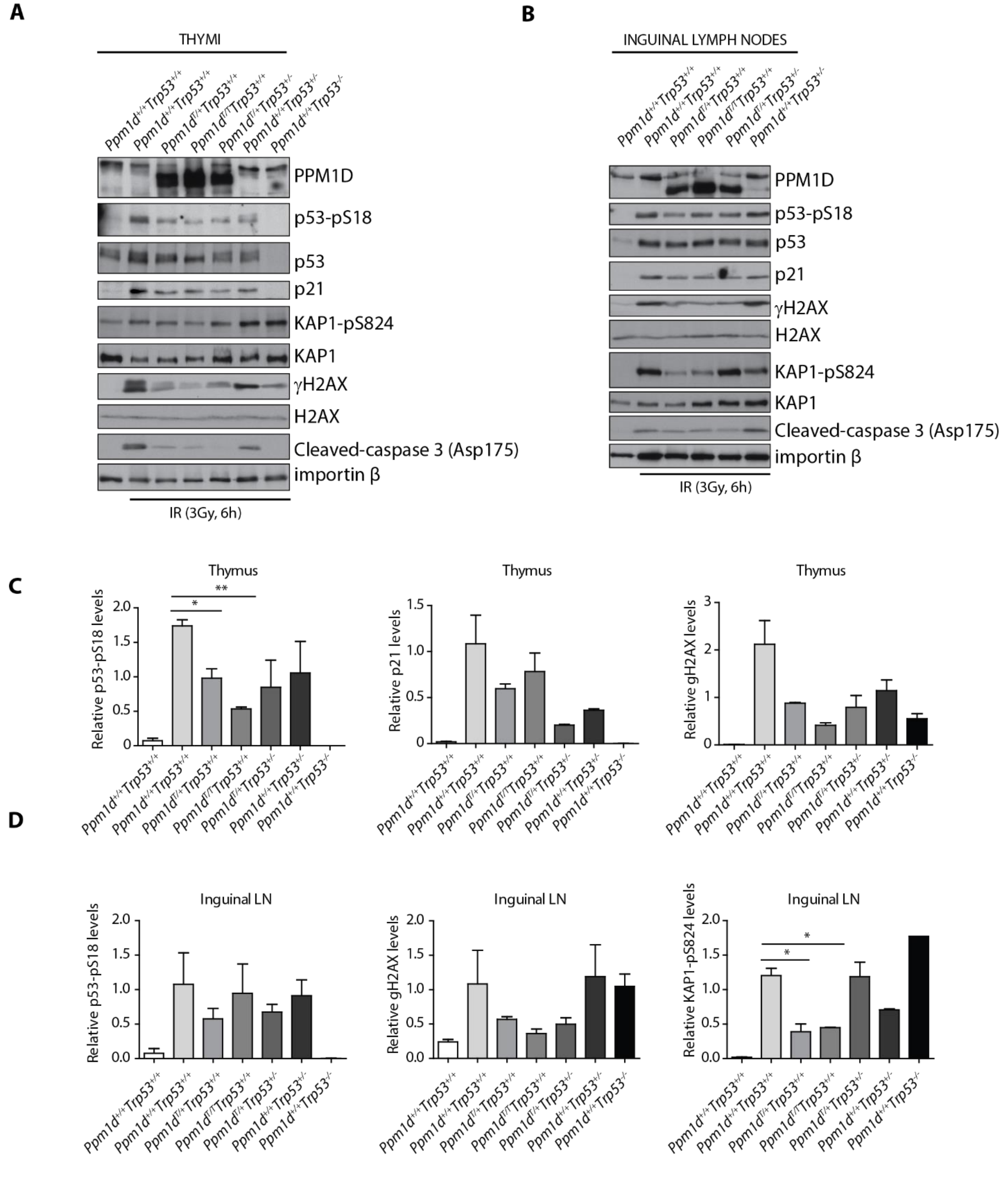

Suppl. Figure S1. Truncated PPM1D impairs DNA damage response

(A) Mice of indicated genotypes were exposed or not to 3 Gy of IR and proteins in the thymi were analyzed after 6 h by immunoblotting. Independent biological replicate of the experiment shown in Fig. 1 F.

(B) Mice of indicated genotypes were exposed or not to 3 Gy of IR and proteins in the inguinal lymph nodes were analyzed after 6 h by immunoblotting. Independent biological replicate of the experiment shown in Fig. 1 G.

(C) Quantification of signal intensity of protein levels in the thymi. Signal was normalized to importin β, (n=3).

(D) Quantification of signal intensity of protein levels in the lymph nodes. Signal was normalized to importin β, (n=3).

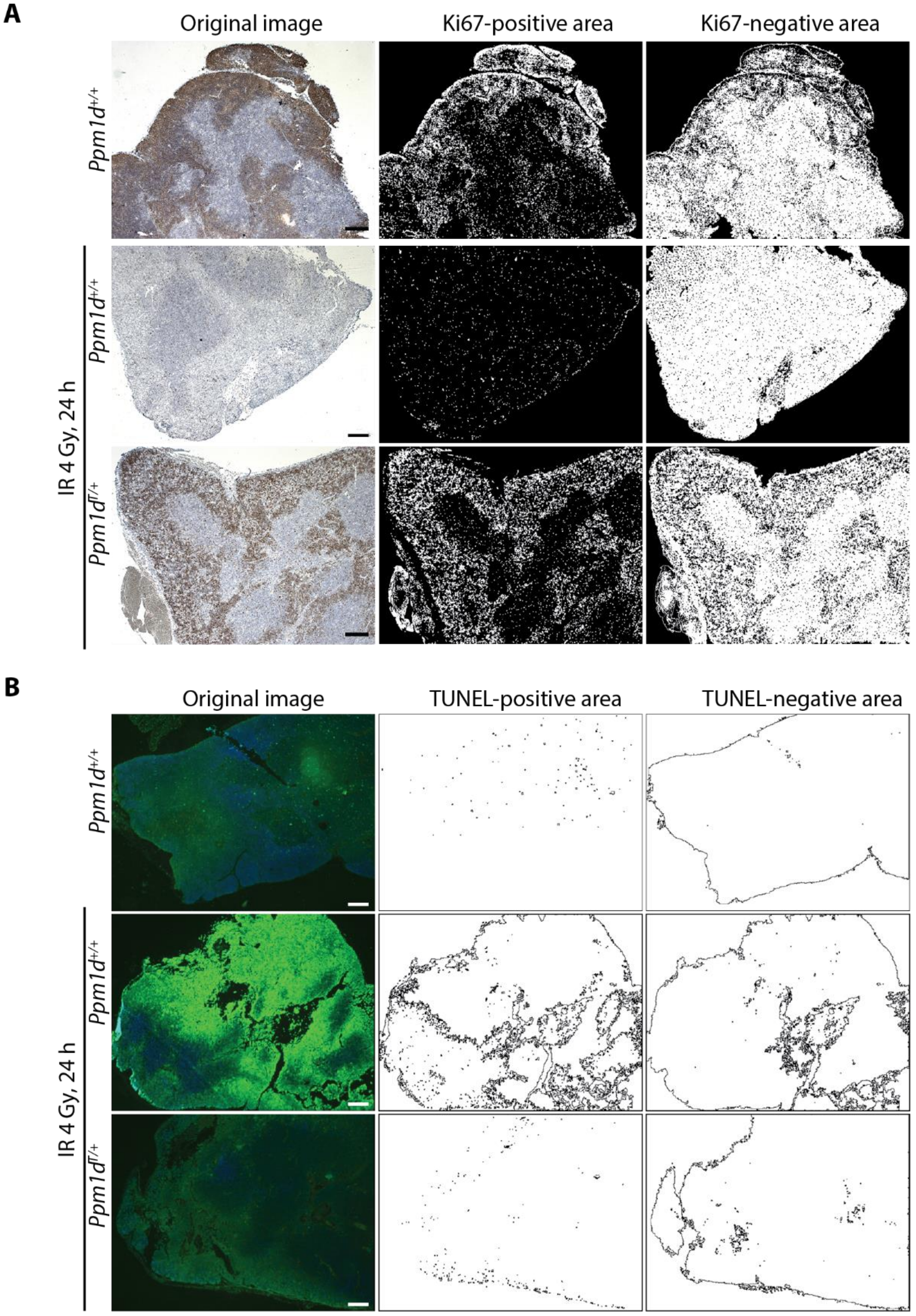

Suppl. Figure S2. Image thresholding for quantification of proliferation and apoptosis in thymus

(A) A representative example of the image thresholding for the proliferation marker Ki-67 positive and negative signal from Fig 2 B. Scale bars indicate 200 μm.

(B) A representative example of the image thresholding for the apoptosis marker TUNEL positive and negative signal from Fig 2 D. Scale bars indicate 200 μm.

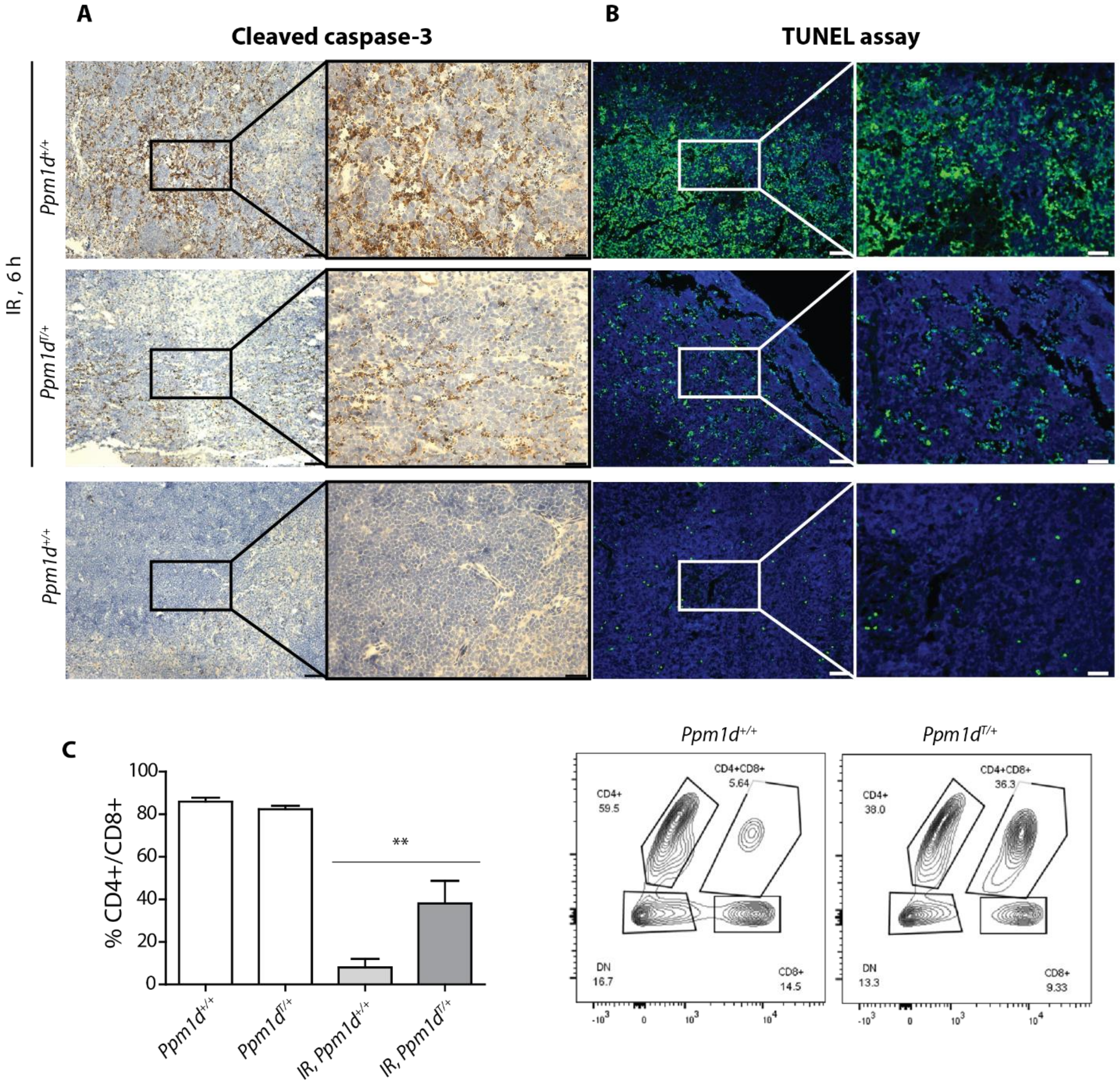

Suppl. Figure S3. Truncated PPM1D prevents activation of caspase 3 after genotoxic stress.

(A) Wild-type and *Ppm1d*$^{T/+}$ mice were sacrificed 6 h after exposure to mock or to ionizing radiation and analyzed by immunohistochemistry. Sections were probed using an antibody against cleaved caspase-3 Asp175. Magnification 20X and 40X, bars indicate 50 μm and 25 μm, respectively. Representative images are shown.

(B) Histology sections of thymi from A were subjected to TUNEL assay. Magnification 20X and 40X, bars indicate 50 μm and 25 μm, respectively.

(C) Wild-type and *Ppm1d*$^{T/+}$ mice were sacrificed 24 h after exposure to mock or to ionizing radiation and population of double-positive CD4+/CD8+ cells was quantified in the thymi using flow cytometry. Representative plot (right panels) and quantification (left panel) is shown. Statistical significance was evaluated by a two-tailed t-test (n=3). Note high difference in the amount of CD4+/CD8+ population between the two genotypes after exposure to IR.

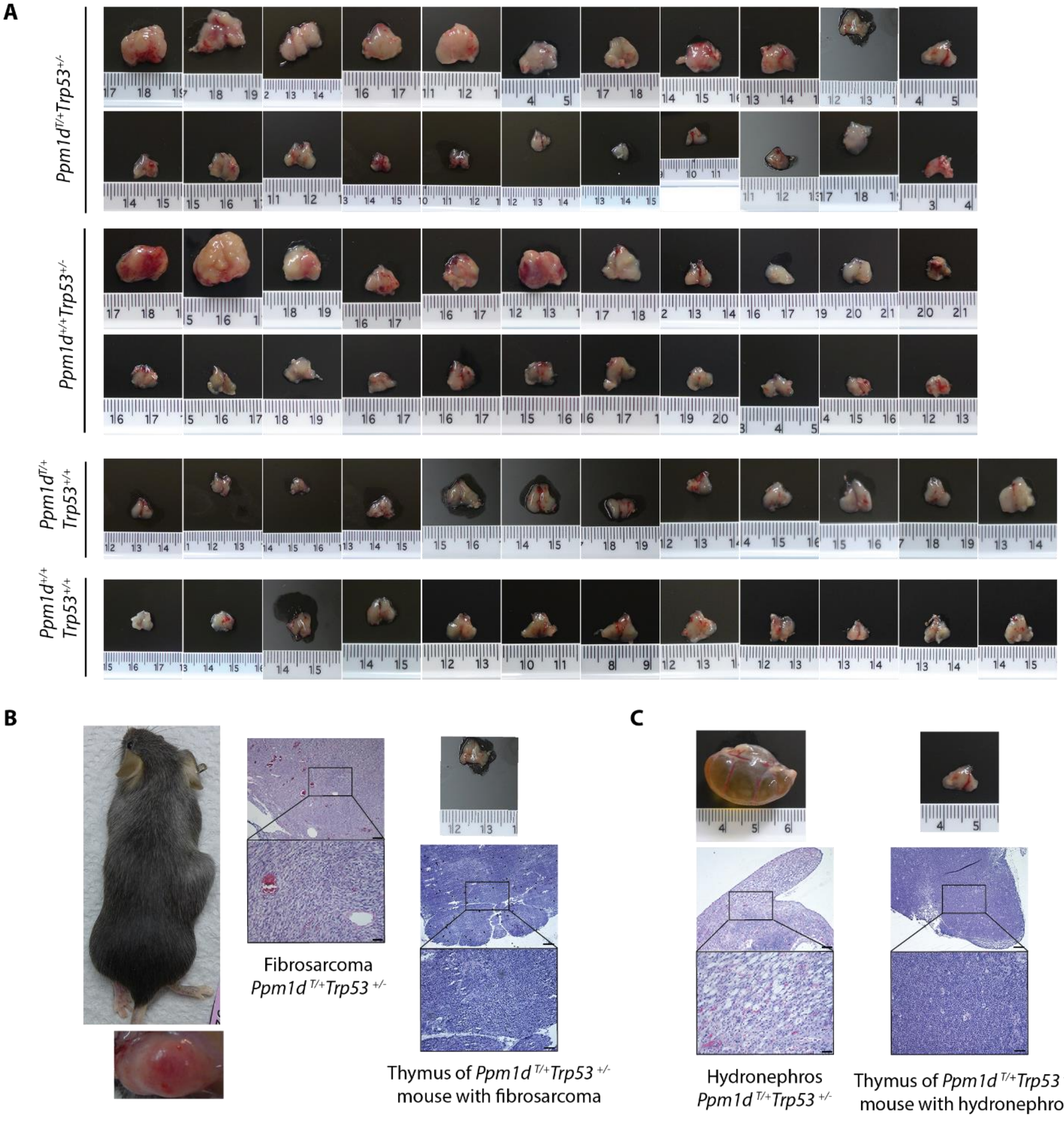

Suppl. Figure S4. Truncated PPM1D promotes tumorigenesis in $Trp53^{+/-}$ background

(A) Images of thymi of mice analyzed in Figure 3. $Ppm1d^{T/+}Trp53^{+/-}$ (N=22); $Ppm1d^{+/+}Trp53^{+/-}$ (N=22); $Ppm1d^{+/+}Trp53^{+/-}$ (N=12); $Ppm1d^{+/+}Trp53^{+/+}$ (N=12).

(B) Image of the $Ppm1d^{+/+}Trp53^{+/-}$ mouse from Fig. 3 carrying a fibrosarcoma (left). Tumor (middle) and thymus (right) were stained by H&E. Scale bars 200 µm (upper panels) and 50 µm (lower panels).

(C) Image of the $Ppm1d^{+/+}Trp53^{+/-}$ mouse from Fig. 3 carrying a hydronephros. Tumor (left) and thymus (right) were stained by H&E. Note impaired medulla/cortex delineation in the thymus. Scale bar 200 µm (upper panel) and 50 µm (lower panel).
